# Protein Degradation Pathways Regulate the Functions of Helicases in the DNA Damage Response and Maintenance of Genomic Stability

**DOI:** 10.3390/biom5020590

**Published:** 2015-04-21

**Authors:** Joshua A. Sommers, Avvaru N. Suhasini, Robert M. Brosh

**Affiliations:** 1 Laboratory of Molecular Gerontology, National Institute on Aging, National Institutes of Health, NIH Biomedical Research Center, 251 Bayview Blvd, Baltimore, MD 21224, USA; E-Mail: sommersj@mail.nih.gov; 2 Department of Medicine, Division of Hematology & Medical Oncology, The University of Texas Health Science Center at San Antonio, San Antonio, TX 78229, USA; E-Mail: avvaru@uthscsa.edu

**Keywords:** helicase, DNA damage response, proteasome, ubiquitin, phosphorylation, acetylation, post-translational modification, Bloom’s syndrome, Fanconi Anemia, Cockayne syndrome, Werner syndrome

## Abstract

Degradation of helicases or helicase-like proteins, often mediated by ubiquitin-proteasomal pathways, plays important regulatory roles in cellular mechanisms that respond to DNA damage or replication stress. The Bloom’s syndrome helicase (BLM) provides an example of how helicase degradation pathways, regulated by post-translational modifications and protein interactions with components of the Fanconi Anemia (FA) interstrand cross-link (ICL) repair pathway, influence cell cycle checkpoints, DNA repair, and replication restart. The FANCM DNA translocase can be targeted by checkpoint kinases that exert dramatic effects on FANCM stability and chromosomal integrity. Other work provides evidence that degradation of the F-box DNA helicase (FBH1) helps to balance translesion synthesis (TLS) and homologous recombination (HR) repair at blocked replication forks. Degradation of the helicase-like transcription factor (HLTF), a DNA translocase and ubiquitylating enzyme, influences the choice of post replication repair (PRR) pathway. Stability of the Werner syndrome helicase-nuclease (WRN) involved in the replication stress response is regulated by its acetylation. Turning to transcription, stability of the Cockayne Syndrome Group B DNA translocase (CSB) implicated in transcription-coupled repair (TCR) is regulated by a CSA ubiquitin ligase complex enabling recovery of RNA synthesis. Collectively, these studies demonstrate that helicases can be targeted for degradation to maintain genome homeostasis.

## 1. Introduction

DNA helicases are a class of enzymes that unwind structured deoxyribonucleic acids in an ATP-dependent manner by translocating in a directionally specific manner (5' to 3' or 3' to 5' with respect to the strand the helicase is predominantly bound), and play essential roles in genome integrity [1,2,3,4,5,6,7]. DNA helicases are classified according to their amino acid conservation within the helicase core domain that is implicated in DNA binding and nucleoside triphosphate hydrolysis. It is estimated that there are 95 helicases or putative helicases (31 DNA helicases and 64 RNA helicases) encoded by the human genome [8]. In addition to their function in catalytically separating complementary strands of double helical DNA by disrupting hydrogen bonds between base pairs, specialized helicases can anneal complementary strands or resolve alternative DNA structures such as triple helices or G-quadruplexes that arise from certain sequence elements. Helicases unwind or branch-migrate structured nucleic acids by several distinct mechanisms which rely on their assembly state (monomer, dimer, tetramer, hexamer, *etc.*) and conformational changes in protein structure induced by nucleotide binding and hydrolysis. Certain helicases can use their motor ATPase function to displace proteins bound to DNA [9,10] or bypass DNA-bound proteins [11]. Evidence from many laboratories has been used to construct working models of how DNA helicases perform their instrumental roles to affect replication, DNA repair, recombination, gene expression, telomere metabolism, sister chromatid cohesion, and chromosome segregation. Accordingly, a number of hereditary disorders are caused by mutations in helicase genes. Of these, DNA helicases implicated in the DNA damage response are particularly prominent with roles in DNA damage sensing, fork maintenance, replication restart, and DNA repair. Overall, it is apparent that helicases operate with a variety of other DNA-metabolizing proteins in a complex myriad of pathways that are vital to maintain chromosomal stability and cellular homeostasis.

It has become increasingly evident that the regulation of helicase function occurs at multiple levels. The expression of helicase genes is up-regulated in a number of cancers, presumably to help cope with the abundance of replicative lesions that arise in rapidly proliferating cells [2]. Interactions of DNA helicases with other proteins can result in an increased or decreased level of DNA unwinding. For example, the single-stranded DNA binding protein Replication Protein A (RPA) physically binds to a number of DNA helicases and stimulates their strand separation activity [12]. On the other hand, the tumor suppressor p53 can inhibit unwinding or branch-migration catalyzed by certain DNA helicases like BLM and WRN defective in Bloom’s syndrome and Werner syndrome, respectively [13,14]. Another mechanism of helicase modulation occurs through post-translational covalent modifications of helicase proteins including phosphorylation, acetylation, SUMOylation, and ubiquitylation (for review, see [15,16]). Such protein modifications can alter the catalytic efficiency of helicase-catalyzed DNA unwinding in either a positive or negative manner. In certain cases, the post-translational modification of a helicase protein may result from a cascade of checkpoint signaling events when cells are exposed to an agent that imposes DNA damage or replication stress. Post-translational modifications can also affect the target protein’s subcellular localization. This may be particularly important for a helicase that is recruited to DNA repair foci.

**Figure 1:**
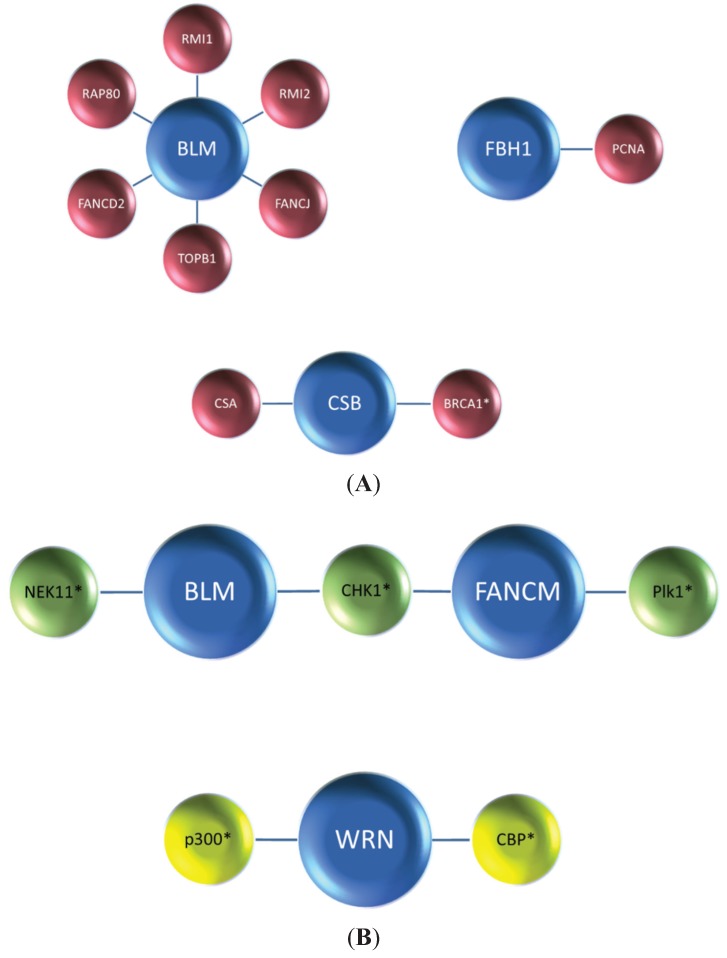
Proteolytic degradation of DNA helicases and helicase-like proteins. Protein interactions (**A**) and post-translational modifications (**B**) of DNA helicases or helicase-like proteins affect their stability. In a number of cases, protein interactions or post-translational modifications of helicase proteins affect their ubiquitylation which in turn influences stability *via* a proteasome degradation pathway. Post-translational modification of helicase proteins by ubiquitylating enzymes are listed in Table 1. See text for details. Blue, helicase or helicase-like protein; Maroon, helicase-interacting DNA repair and/or replication protein; Green, protein kinase; Yellow, acetyltransferase. The asterisk in *Panel A* indicates that BRCA1 has an intrinsic ubiquitin ligase activity. The asterisks in *Panel B* indicate the involvement of phosphorylation by protein kinases (NEK11, CHK1, PlK1) or acetylation by acetyltransferases (p300, CBP) in helicase protein stability.

Yet another mechanism for modulation of helicase function is mediated by protein degradation pathways. In this scenario, the very stability of DNA helicase or helicase-like proteins is affected by cellular responses to stress that cause their proteolytic degradation. Regulation of proteins implicated in the DNA damage response or replication *via* their degradation is not unprecedented. For example, in yeast, proteasome degradation of replisome proteins regulates genomic stability [17]. However, much less is known about the intricacy of genome maintenance pathways and how they are regulated by proteasome degradation in higher eukaryotes. This review will provide a unique perspective on the topic of mammalian helicase protein degradation pathways to inform the reader of the emerging mechanisms that cells use to regulate helicase-dependent DNA repair, checkpoint signaling, and gene expression. Typically, helicase protein interactions play a major role in conferring helicase protein stability (Figure 1A), and the degradation of DNA helicases is frequently mediated by a ubiquitin-proteasome system in which the ubiquitin ligase complexes responsible for signaling proteasomal degradation have been identified (Table 1). In some cases, post-translational modifications such as phosphorylation or acetylation are involved (Figure 1B). We will discuss examples of helicase degradation pathways with a focus on human DNA helicases implicated in the cellular response to DNA damage or replication stress. Collectively, the evidence suggests that helicase degradation is an important regulatory mechanism which may be under-appreciated. Understanding helicase degradation pathways is likely to provide important insights to molecular-genetic diseases and potential avenues for therapy.

## 2. Ubiquitin-Proteasome Pathway of Protein Degradation

Pathways of protein degradation are for the most part governed by post-translational modifications, a rapidly growing field of study and interest. Proteolytic digestion of key proteins in the DNA damage response is highly regulated and can occur in a timely fashion [18,19,20]. DNA damage signaling is often initiated by protein phosphorylation by cellular kinases which can facilitate recruitment of protein targets to DNA damage foci or stalled replication forks. DNA damage response proteins can be degraded by ubiquitin-proteasome pathways to enable a cascade of cellular events influencing genome stability and cellular homeostasis. The ubiquitin-proteasome system is responsible for the degradation of short-lived or abnormal proteins in eukaryotic cells. The ubiquitin modification itself involves the covalent addition of a 76 amino acid ubiquitin protein to a primary amine of the target protein. Target proteins can be mono-ubiquitylated or poly-ubiquitylated, which provides information for several different fates: degradation, conformational alteration, or specific protein interaction. The 26S proteasome is responsible for degradation *via* the ubiquitin pathway. Protein degradation which occurs as a component of the DNA damage response is highly relevant to DNA helicases. In the following sections, we will focus our discussion on recently characterized proteins and events involved in DNA helicase degradation.

**Table 1:** Helicase or helicase-like proteins modified by ubiquitylating ligases.

Helicase	Ubiquitylating Enzyme	Effect on Helicase	Reference
BLM	RNF8/RNF168	relocalization	[21]
	MlB1	degradation	[22]
	CUL3	degradation	[23]
	E1b55K/E4orf6	degradation	[24]
FANCM	SCF^β-TRCP^	degradation	[25]
FBH1	CRL4^Ctd2^	degradation	[26]
HLTF	CHFR	degradation	[27]
CSB	CUL4A/CSA	degradation	[28]
	BRCA1	degradation	[29]

## 3. Control of Bloom’s Syndrome Helicase (BLM) Protein Level and Localization

The Amor-Gueret laboratory made one of the first observations that expression of the RecQ DNA helicase defective in Bloom’s syndrome (BLM) is regulated when they analyzed the level of BLM protein by immunoblotting of extracts from cycling human cervical cancer (HeLa) cells either untreated or treated with the replication inhibitors hydroxyurea (HU) or aphidicolin (APH) [30]. In untreated cells, they found that BLM protein was hardly detectable in G1, but enriched in S and G2/M phases. In those cells treated with a replication inhibitor, BLM accumulated in S phase. Exposure to a microtubule-disrupting drug that arrests cells at G2/M resulted in a slower migrating form of BLM as observed by SDS-PAGE analysis, suggesting that BLM is post-translationally modified during mitosis. Restoration of immunoprecipitated BLM from mitotic cells to its normal migration by phosphatase treatment indicated that BLM is phosphorylated.

**Figure 2:**
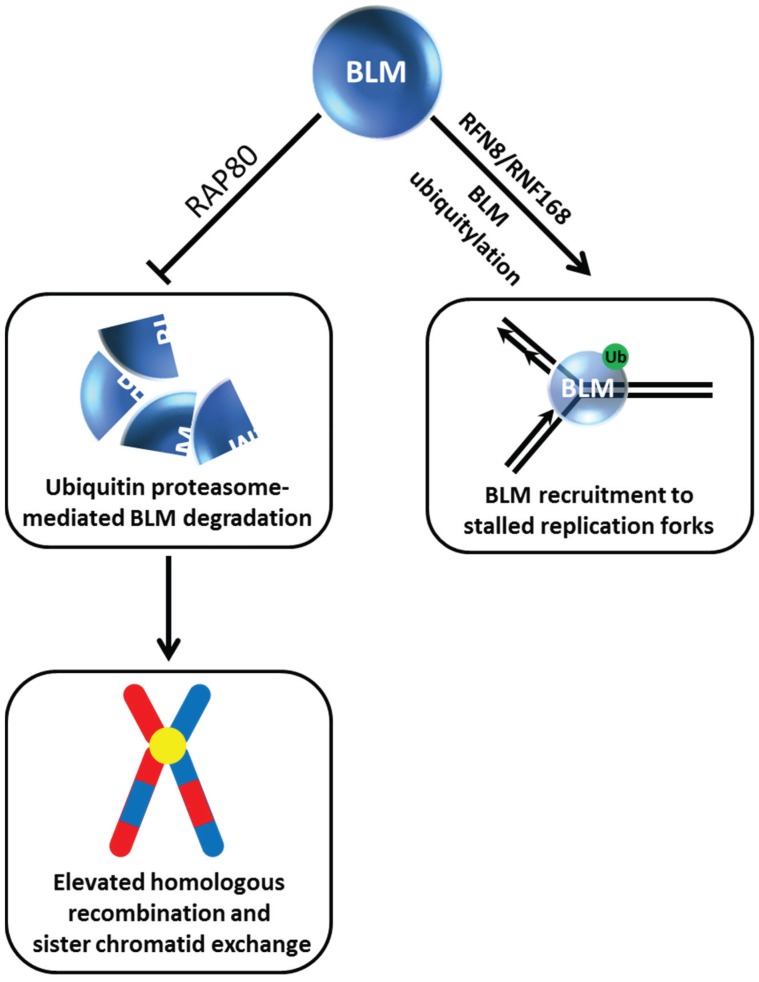
Dynamics of BLM helicase ubiquitylation govern its stability and recruitment to stalled replication forks and suppression of sister chromatid exchange. See text for details.

Evidence over the past decade indicates that BLM post-translational modifications including phosphorylation, ubiquitination, and SUMOylation regulate its pro- and anti-recombinogenic functions dictating its roles in chromosomal stability (for review, see [15]). The importance of BLM post-translational modifications for BLM protein stability and subcellular localization is coming to light as well. In recent work, the Sengupta laboratory reported that BLM is recruited to HU-induced replication stress foci in a manner dependent on its ubiquitylation by the E3 ligase RNF8/RNF168 [21] (Figure 2). In the absence of stress, RFN8-ubiquitylation of BLM is required for its proper subcellular localization to the nucleoplasm and promyelocytic leukemia (PML) nuclear bodies. The ubiquitin-interacting motifs adaptor protein RAP80 was determined to be responsible for recruitment of BLM to stalled replication foci, and this localization is necessary for BLM suppression of homologous recombination (HR) at stalled forks to help minimize sister chromatid exchange (SCE) (Figure 2). RAP80 serves an additional purpose to preserve the stability of BLM in unstressed cells. A deficiency in RAP80 causes BLM to be degraded by a ubiquitin-proteasomal pathway, resulting in an elevated level of HR and SCE. The identity of the ubiquitin-ligase mediating BLM degradation in the RAP80-regulated pathway is yet to be determined.

Regulation of the sub-nuclear localization of BLM is complex and likely to be controlled at multiple levels. Very recently, the Bernstein laboratory reported that a deficiency in the SUMO-targeted ubiquitin ligase RNF4 in untreated human osteosarcoma (U-2 OS) cells increased BLM foci which co-localized with PML nuclear bodies [31]. It remains to be determined if RNF4 status influences BLM stability in addition to its subcellular localization. As will be discussed below, BLM protein interactions as well as post-translational modifications dramatically affect cellular BLM protein levels.

## 4. Fanconi Anemia Protein Complex Controls BLM Degradation and Its Role in the DNA Damage Response and Maintenance of Genomic Stability

Despite the initial findings that BLM phosphorylation status does not dictate its stability in a strictly cell cycle regulated proteasomal-dependent manner, subsequent studies showed that BLM stability and its ability to form DNA damage-induced foci is regulated by its protein partners. Studies in chicken DT40 cell lines provided early experimental data suggesting interplay of BLM with the Fanconi Anemia (FA) pathway important for the response to interstrand cross-links (ICL) and maintenance of genomic stability [32]. Elevated SCE, a hallmark of BLM-deficient cells, was observed in *fancc* cells, but the SCE level was the same in *blm* cells as *blm fancc* cells suggesting that FANCC and BLM operate in the same pathway. Chicken or human *fancc* or *fancd2* cells transfected with a plasmid encoding BLM protein conjugated to Green Fluorescence Protein (GFP) were dramatically depressed in their ability to form GFP-BLM foci after cellular exposure to the DNA cross-linking agent mitomycin C (MMC). These findings were significant because they suggested that the FA pathway controls BLM trafficking and its functionality in HR and the ICL response. As it would turn out, BLM’s interactions with its protein partners in the FA pathway play a critical role in its stability as well (see next section).

**Figure 3:**
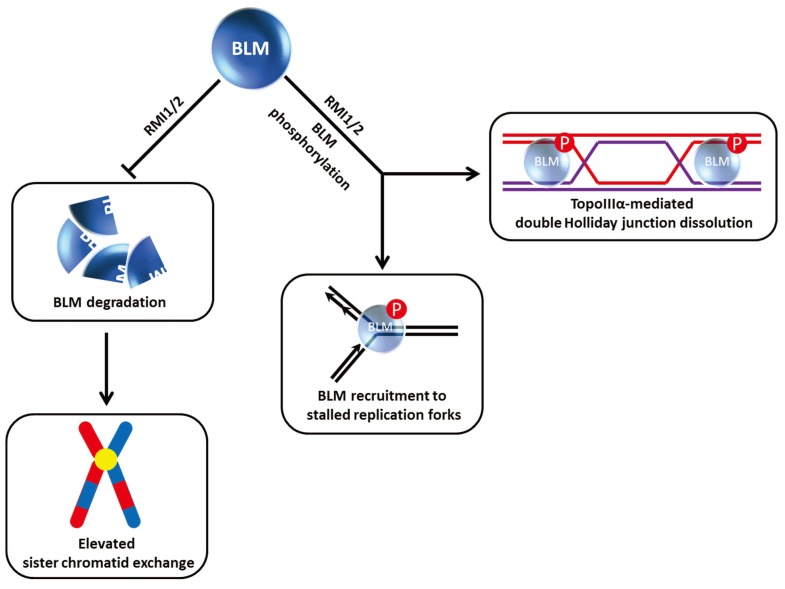
RMI1/2 is important for BLM stability, phosphorylation, and its role in double Holliday Junction dissolution. See text for details.

Studies from the Wang laboratory identified a multi-protein BLM complex that is important for genome integrity [33]. Among its components, the oligonucleotide-binding (OB)-fold domain protein RMI1 (also known as BLAP75) was found to be important for BLM and Topoisomerase III alpha (TopoIIIα) stability as demonstrated by immunoblot analysis of nuclear extracts from cells depleted of RMI1 by RNA interference [34] (Figure 3). Like FANCC and FANCD2, RMI1 deficiency resulted in the impaired recruitment of BLM to DNA damage foci. Harkening back to observations that BLM is hyper-phosphorylated during mitosis [30], RMI1 depletion interfered with BLM phosphorylation during mitosis and also decreased cell proliferation [34] (Figure 3). Moreover, the RMI1-depleted cells showed elevated SCE, consistent with its importance as a key stabilizing factor of the BLM complex. Another OB-fold domain protein known as RMI2 is also found in the BLM complex and required for BLM protein stability as well [35]. The RMI complex, consisting of RMI1 and RMI2, stimulates BLM-TopoIIIα double Holliday Junction dissolution, and helps to suppress SCE through its association with the BLM complex (Figure 3).

Collectively, the experimental evidence suggests that cooperativity of the FA pathway with BLM is mediated by a super-complex of proteins that include BLM, RPA, TopoIIIα, RMI1/2, FANCM, and members of the FA core complex (for review, see [36,37]). Two separate domains in FANCM mediate protein interactions of BLM with the FA core complex (*via* direct contact with FANCF) and TopoIIIα (*via* direct contact with RMI1 and TopoIIIα) [38]. Based on deletion mapping and genetic complementation studies, a model was proposed for the interactive role of the FA complex with the BLM dissolvasome to maintain a normal level of SCE and to repair ICLs by a HR repair pathway. Given that RMI1 and RMI2 manage BLM protein level and stability of the BLM super-complex, modulation of BLM physical interactions with other proteins by small molecules may provide an opportunity to induce lethality of cancer cells that incur replicative lesions leading to defective replication restart and unrepaired toxic double strand breaks (DSBs). Advances in the characterization of the molecular structure of protein interactions defining the BLM complex (for review, see [39]) may be useful for *in silico* docking simulations with virtual small molecule libraries.

## 5. FANCJ and FANCD2 Regulate BLM Stability and Function

**Figure 4:**
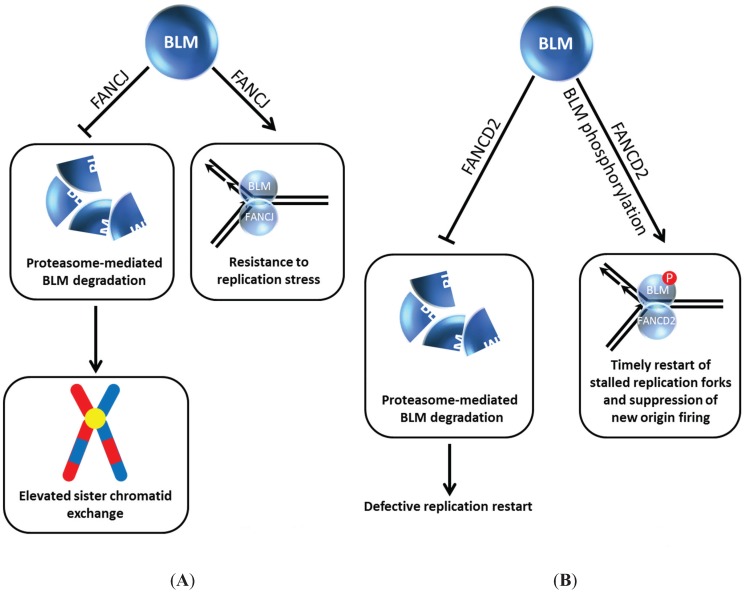
BLM interaction with FANCJ (**A**) or FANCD2 (**B**) prevents its degradation and provides resistance to replication stress and timely restart of stalled replication forks. See text for details.

Our own work demonstrated that the interplay between BLM and the FA pathway extends beyond FANCM and members of the upstream FA core complex. We determined that BLM directly binds to the FANCJ helicase (Figure 4A), the latter proposed to act in HR repair through its interactions with BRCA1 [40] and MRE11 [41]; moreover, FANCJ and BLM helicases acting together synergistically unwind a damaged DNA substrate [42]. In human cells, BLM protein level was dramatically reduced in FANCJ-depleted cells within 24 h after siRNA-FANCJ transfection. The negative effect of FANCJ deficiency on BLM protein level was confirmed using extracts from an isogenic pair of FANCJ mutant and corrected cell lines. Strikingly, the effect was specific as the levels of the BLM-interacting partners TopoIIIα, Flap Endonuclease 1 (FEN-1), and RPA were unaffected. Moreover, the effect was not reciprocal, *i.e.*, FANCJ protein was not affected by BLM status, as demonstrated by immunoblot analysis of extracts from an isogenic pair of BLM mutant and corrected cells. The adverse influence of FANCJ deficiency on BLM protein level was not mediated at the transcriptional level; furthermore, normal BLM protein level was restored in FANCJ-depleted cells by exposing the cells to the proteasome inhibitor MG132. Thus, FANCJ stabilizes BLM protein level by preventing its proteasomal degradation (Figure 4A). The BLM interaction domain was mapped to the C-terminal region of FANCJ [42], which overlaps with the BRCA1-binding site (phosphorylated Ser-990) [43] and the Topoisomerase IIβ binding protein 1 (TopBP1)-binding site (phosphorylated Thr-1133) [44]. Therefore, it will be important to ascertain if phosphorylation of FANCJ Ser-990 or Thr-1133 governs the BLM interaction as it does for the BRCA1 and TopBP1 interactions, respectively.

A physiological importance of the BLM-FANCJ interaction is supported by several lines of experimental evidence: (1) BLM and FANCJ co-localize in discrete foci after cellular exposure to the replication stress inducing agents MMC or HU; (2) FANCJ-deficient cells and BLM-deficient cells share overlapping phenotypes including elevated SCE and sensitivity to agents that induce replication stress; (3) expression of a BLM-interacting C-terminal domain fragment of FANCJ exerts a dominant negative effect on HU resistance of human cells [42]. An important area of research is to assess if BLM deficiency in FA-J patients potentially contributes to apparent clinical heterogeneity that is observed in individuals with FANCJ mutations [45,46]. This issue is not only relevant for the FANCJ-BLM interaction, but likely other helicase partnerships as well where the stability of the interacting protein(s) is affected. In terms of the FANCJ-BLM interaction, future efforts will likely address how the two helicases coordinate to enable an adequate and timely response to stalled replication forks or DSBs [47].

FANCI and FANCD2, key signaling molecules of the FA pathway which become activated upon DNA damage induced mono-ubiquitylation, play a critical but still not well understood role in the response to ICL-induced DNA damage [48]. The Sobeck laboratory showed that immunodepletion of FANCD2 from Xenopus egg extracts resulted in destabilization of BLM, and this was independent of FANCI, another FA signaling molecule that is recruited to stalled replication forks [49] (Figure 4B). Degradation of BLM in FANCD2-depleted egg extracts is prevented by the inclusion of proteasome inhibitor MG132 in the extract preparation. In a key experiment, the authors reconstituted FANCD2-depleted extracts with purified recombinant myc-tagged FANCD2 and showed that the BLM protein level was stabilized, indicating that a deficiency in FANCD2 protein itself and not a co-depleted protein caused the destabilization of BLM. The destabilizing effect of FANCD2 deficiency on BLM was found to be evolutionarily conserved, as demonstrated by studies using an isogenic pair of patient-derived FANCD2 mutant and corrected cells [49]. In DNA fiber tract analysis with human cells experiencing replication stress, it was determined that BLM and FANCD2 collaborate to promote restart of stalled forks and suppress firing of new replication origins (Figure 4B).

Returning to the Xenopus studies, incubation of egg extracts with double-stranded DNA fragments resulted in BLM hyper-phosphorylation in a FANCD2-dependent manner [49] (Figure 4B), consistent with the idea that *in vivo* BLM becomes hyper-phosphorylated when cells experience DNA damage. The dependence on FANCD2 for BLM phosphorylation is akin to its dependence on RMI1 [34]; however, if the BLM phosphorylation sites and kinases involved are the same or not remains to be seen. Moreover, further work is required to ascertain if BLM phosphorylation status is directly related to its protein stability, *i.e.*, if hypo-phosphorylated BLM is less stable than hyper-phosphorylated BLM.

**Figure 5:**
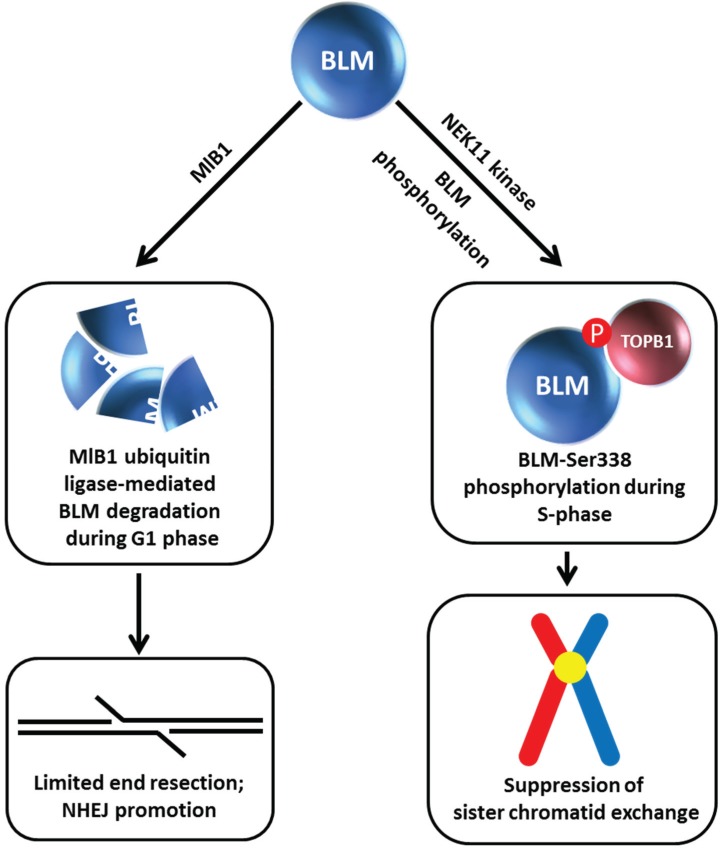
MlB1 ubiquitin ligase and NEK11 kinase influence BLM stability which has outcomes for non-homologous end-joining and suppression of sister chromatid exchange. Certain aspects of this model are debated. See text for details.

## 6. BLM Phosphorylation and Ubiquitin Ligase-Mediated BLM Degradation

Given the apparent importance of BLM protein interactions for BLM stability, understanding the molecular mechanisms and cellular pathways involved is an active area of investigation. Wang *et al.* discovered a cell cycle-regulated interaction between BLM and TopBP1 mediated by the 5th BRCT phosphoprotein-binding domain (BRCT5) in TopBP1 [22] (Figure 5). Mutation of Ser-338 in the N-terminal region of BLM (which is phosphorylated specifically during S-phase) strongly disrupted the BLM/TopBP1 interaction. However, in contrast to the Wang *et al.* study [22], Blackford *et al.* determined that phosphorylation of Ser-304 of BLM mediates the interaction with TopBP1; furthermore, disruption of the TopBP1-BLM interaction did not destabilize BLM protein [50]. Studies by Wang *et al.* showed that the S-phase specific protein kinase NEK11 is responsible for BLM-Ser-338 phosphorylation, and that depletion of NEK11 kinase significantly reduced BLM protein level [22] (Figure 5). Consistent with this observation, TopBP1 depletion dramatically decreased cellular BLM protein levels, and in turn, resulted in elevated SCE. Ectopic expression of wild-type TopBP1, but not a TopBP1 mutant lacking BRCT5, restored normal BLM protein level and reduced SCE. Although Blackford *et al.* observed that neither a deficiency in TopBP1 nor perturbation of the TopBP1-BLM interaction affected stability of BLM protein, they did observe that the interaction between BLM and TopBP1 is important to maintain genomic stability [50]. In another study, TopBP1 was found to interact with FANCJ [44], which was aforementioned to affect BLM stability [42]. However, expression of a TopBP1 mutant protein lacking BRCT domains 7 and 8, which mediate the interaction with FANCJ [44], restored normal BLM protein level and SCE [22], indicating a FANCJ-independent effect of TopBP1 on BLM protein stability and its role in genome maintenance through suppression of cross-overs.

Similar to what was observed for the BLM-FANCJ relationship, treatment of TopBP1-depleted cells with the proteasome inhibitor MG132 restored BLM protein level [22]. Wang *et al.* found that BLM interacts with the E3 ubiquitin ligase MlB1 in a phosphorylation-dependent manner *via* its helicase domain, and that MlB1 ubiquitylates BLM *in vivo* and *in vitro* (Figure 5). *In vitro* studies mapped lysine residues K38, K39, and K40 in the N-terminal region of BLM as the potential E3 ubiquitylation sites. The authors next wanted to ascertain if the effects of TopBP1 and E3 ligase MlB1 on BLM protein stability were coordinated with each other. Overexpression of wild-type TopBP1, but not the TopBP1 mutant lacking BRCT5 and therefore defective in its interaction with BLM, decreased MlB1 binding to BLM, suggesting that BLM engages in a protein interaction with either MlB1 or TopBP1, but not both simultaneously. This mutually competitive relationship was borne out by cellular studies in which it was found that the BLM/MlB1 interaction is detected in G1 phase cells (when BLM is most susceptible to degradation), whereas the BLM/TopBP1 interaction is enriched in S phase. Consistent with this, the BLM triple lysine mutant (K38, K39, K40) displayed increased stability in G1 compared to wild-type BLM. Moreover, BLM protein levels were restored to normal in TopBP1-depleted cells when MlB1 was co-depleted. Based on their results, Wang *et al.* proposed a model in which TopBP1 and E3 ligase MlB1 play antagonistic roles in regulating BLM protein stability. This model is provocative because it suggests that cell cycle-regulated BLM partnerships profoundly influence BLM protein level which consequently influences its roles in genome stability and DNA repair. During S phase TopBP1 acts as a BLM stabilizer so that it can suppress SCE. Conversely, BLM is degraded in G1 by a MlB1-mediated pathway which helps to limit end resection (normally used in an early phase of HR repair during S phase), thereby allowing the nonhomologous end-joining (NHEJ) pathway of DSB repair to take place (Figure 5). However, the authors point out that the MlB1 E3 ubiquitin ligase may have additional protein targets that serve to modulate DNA repair. It should also be pointed out that the role of the three N-terminal lysines of BLM (residues K38, K39, K40) in genome stability is controversial. Blackford *et al.* observed that expression of this triple mutant BLM protein was comparable to unmutated BLM and that the BLM triple lysine mutant displayed a reduced interaction with TopoIIIα RMI1/RMI2, suggesting that either defects in BLM association with other components of the dissolvasome or its inability to be ubiquitylated may contribute to mutant cellular phenotypes [50].

**Figure 6:**
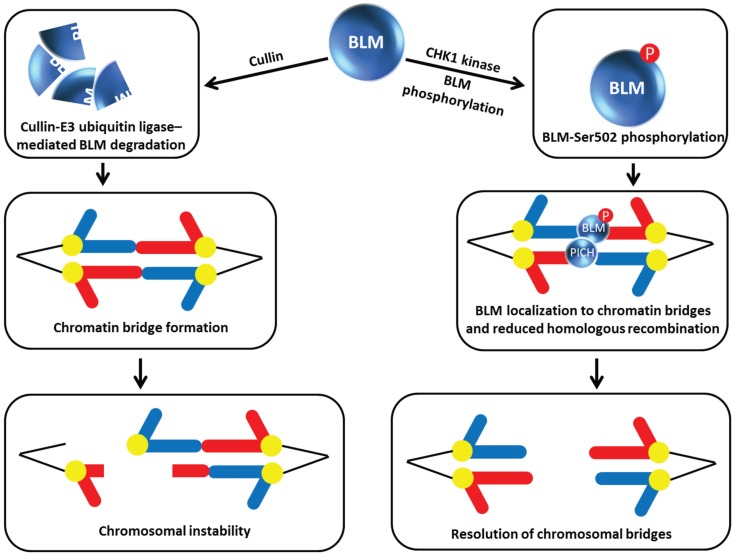
Chk1 phosphorylation of BLM prevents its degradation by Cullin E3 ubiquitin ligase, enabling BLM to collaborate with PICH and resolve chromosomal bridges. See text for details.

In the latest work on how BLM function is regulated by its own degradation, post-translational modification by phosphorylation of a BLM residue that is different from that one targeted by NEK11 (mentioned above, [22]) was discovered. In this case, rather than modulating the protein interaction of BLM with TopBP1, the constitutive phosphorylation of BLM at Ser-502 by checkpoint kinase 1 (Chk1) enables BLM to localize to chromatin bridges where it is believed that the helicase inhibits HR or operates with another helicase known as Polo-like kinase 1-interacting checkpoint helicase (PICH) to unravel chromatin and promote resolution of incompletely segregated chromosomal DNA (*i.e*., bridges) that can form during anaphase [23] (Figure 6). Petsalaki *et al.* performed an elegant set of experiments characterizing BLM site-directed mutants in which Ser-502 was substituted with a non-phosphorylatable alanine (A) or phosphor-mimicking aspartic acid (D). The BLM-S502A mutation reduced BLM stability whereas the BLM-S502D mutation stabilized BLM allowing it to prevent chromatin bridges in Chk1-deficient cells. BLM degradation is mediated by the Cullin E3 ubiquitin ligase complex based on the observation that the two human recombinant proteins expressed in Chinese hamster ovary cells can be pulled down together and that depletion of Cullin inhibited BLM degradation and suppressed the accumulation of anaphase bridges in cells treated with a Chk1 inhibitor. Thus Chk1 phosphorylation of BLM Ser-502 blocks Cullin binding to BLM and prevents its degradation (Figure 6). It has not yet been determined if Chk1 phosphorylation of BLM at residue Ser-502 may affect other functions of BLM besides protection against chromatin bridges through its effect on BLM protein stabilization.

**Figure 7:**
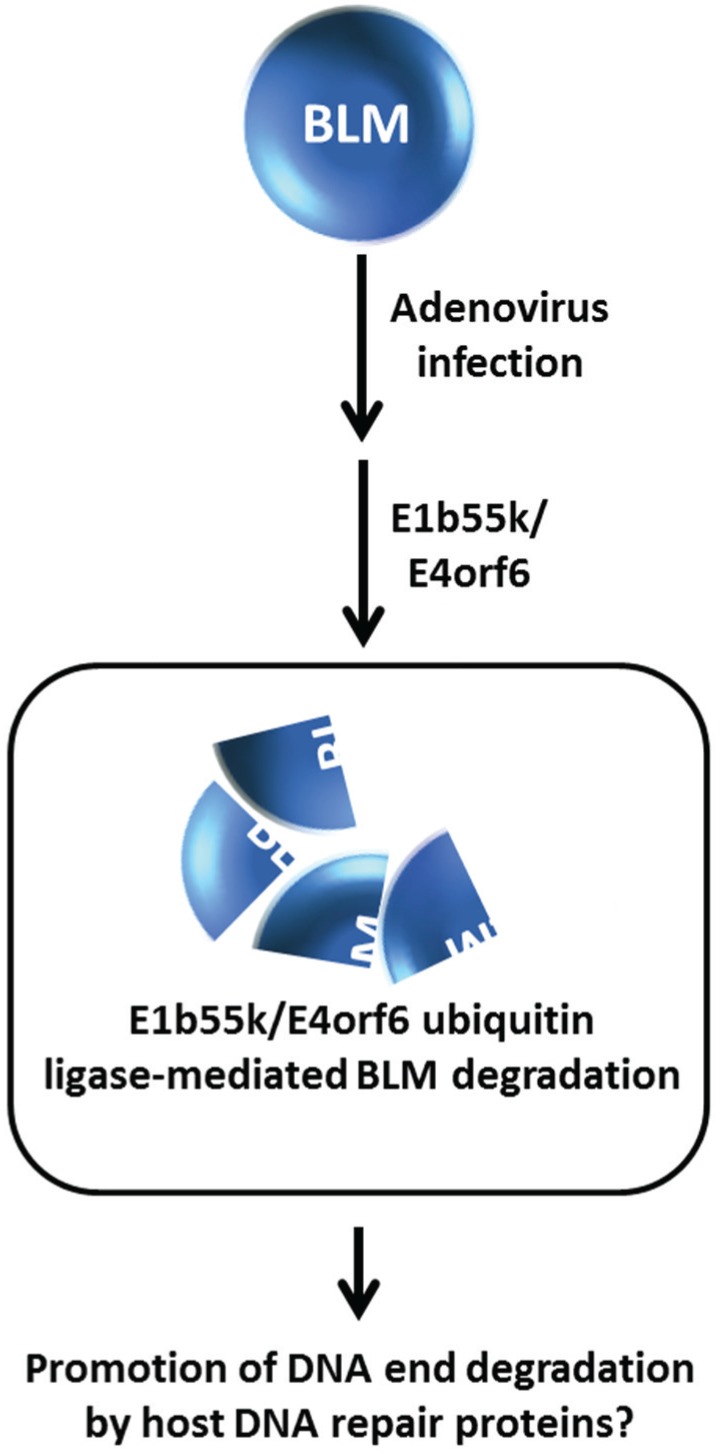
Adenovirus infection promotes E1b55k/E4orf6 ubiquitin ligase-mediated mediated BLM degradation. See text for details.

From a broader perspective, degradation of BLM and other DNA repair or replication response proteins promoted by an E3 ubiquitin ligase may have implications in additional areas other than genome stability maintenance and response to DNA damage induced exogenously by chemical agents or radiation. Viral infection can elicit the recruitment of DNA damage response and repair proteins to sites of viral replication where the host machinery may be either suppressed or exploited to create an optimal environment for infection and proliferation of the virus [51]. The adenovirus E1b55K and E4orf6 gene products constitute an E3 ubiquitin ligase that facilitates degradation of cellular DNA repair proteins such as the tumor suppressor molecule p53 and the Mre11-Rad50-Nbs1 (MRN) complex implicated in DNA DSB recognition and end trimming at sites of DSBs to enable viral infection [52,53]. Given that BLM is implicated in DNA end resection, Orazio *et al.* investigated the effect of adenovirus infection on BLM stability [24]. They determined that BLM is degraded by an E1b55K/E4orf6- mediated proteasome pathway independent of the MRN complex in HeLa cells infected with adenovirus (Figure 7); however, the functional relevance of BLM degradation is unclear. The localization of BLM at sites of viral infection suggests a role of BLM with possibly MRN to process viral DNA ends. Further studies of how human DNA damage response proteins like BLM, or Werner syndrome helicase (WRN) which is a HIV-1 cofactor [54], play a role in viral infection and growth may prove to be informative in the development of anti-viral medicines.

## 7. Modification of FANCM Translocase by Checkpoint Kinases Dictates FANCM Stability and Its Role in Chromosomal Stability and Replication Restart

**Figure 8:**
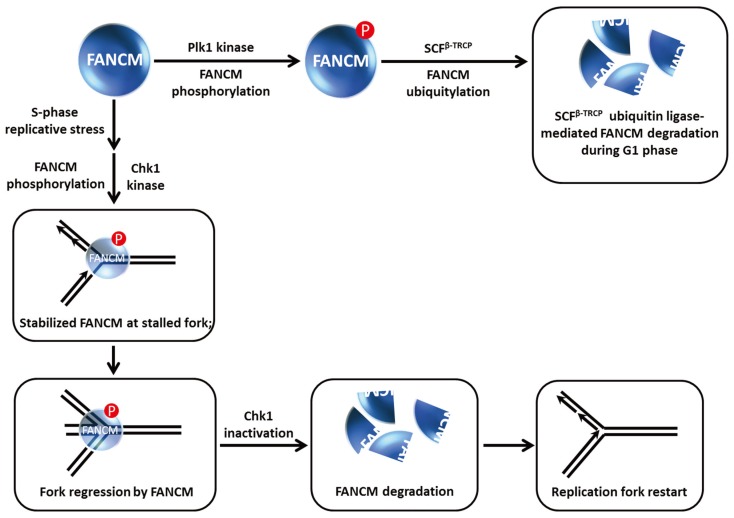
FANCM phosphorylation controls FANCM stability and its role at stalled replication forks. See text for details.

Although not a classic DNA helicase *per se*, FANCM is an ATP-dependent translocase capable of remodeling DNA structures associated with stalled replication forks [55]. FANCM is implicated in the FA pathway of ICL repair, and is more generally involved in the replication stress response. FANCM recruits the FA core complex to chromatin during S phase or the DNA damage response, and as mentioned above acts as a bridging molecule with the BLM protein to maintain chromosomal stability through BLM-TopoIIIα double Holliday Junction dissolution [56]. Work from the D’Andrea laboratory showed that FANCM is hyper-phosphorylated and proteolytically degraded during mitosis [25] (Figure 8). They found that the β-TRCP component of the SCF E3 ubiquitin ligase complex was responsible for FANCM proteolysis mediated by the presence in FANCM of a consensus DSGxxS consensus sequence that is implicated in SCF^β−TRCP^ degradation. The Plk1 kinase which regulates mitotic progression through its checkpoint functions is responsible for FANCM phosphorylation by recognition of a Polo-box domain, which serves to trigger subsequent FANCM degradation. Remarkably, FANCM degradation was shown to facilitate the release of the FA core complex from chromatin during mitosis (independent of its role in S-phase checkpoint), and this is required for the suppression of chromosomal radial formation induced by cellular exposure to the cross-linking agent MMC.

The precise roles of FANCM in the DNA damage checkpoint response are still being investigated. Luke-Glasser *et al.* found that FANCM is necessary for resumption of DNA synthesis after a replication block induced by the topoisomerase inhibitor camptothecin (CPT) [57]. This led the authors to monitor the S-phase checkpoint in FANCM-depleted cells; however, Chk1 phosphorylation appeared to be intact but the level of Chk1 protein was decreased, particularly after CPT, HU or APH treatment. Flipping the coin, FANCM protein level was also decreased after cellular CPT exposure; moreover, FANCM was also destabilized when cells were co-treated with a replication inhibitor (HU, APH) and the Chk1 kinase inhibitor UCN01 or the cells were depleted of Chk1 altogether. It was suggested that Chk1 kinase inhibition may be required to elicit FANCM destabilization in cells exposed to HU or APH *versus* CPT because CPT does not cause strong Chk1 activation in the first place (Figure 8). In accord with the earlier findings that FANCM is degraded by a ubiquitin-proteasome pathway [25,58], Luke-Glasser *et al.* observed that the proteasome inhibitor MG132 enabled cells co-treated with HU and the Chk1 inhibitor UCN01 to retain stabilized FANCM [57].

These findings coupled with the observations that FANCM promotes steady replication fork progression particularly in the context of replication stress and remodels stalled replication forks to prevents single-stranded DNA exposure led Luke-Glasser *et al.* to propose the following model [57]: During situations of replication stress, FANCM catalyzes fork regression, and then promotes coupling of leading and lagging strand synthesis during replication restart; the single-stranded DNA that arises at stalled forks causes Chk1 activation which helps to preserve FANCM from proteolytic degradation so that it can do its job of fork reversal, which may also involve BLM. FANCM and Chk1 stabilize each other in a reciprocal manner. The stabilization of Chk1 by FANCM leads to a stimulation of checkpoint signaling. Ultimately, fork reversal results in loss of the single-stranded DNA signal such that Chk1 is no longer activated; consequently FANCM is degraded as leading and lagging strand synthesis are coupled once again. In the case of replication-blocking lesions like a topoisomerase-inhibitor drug-DNA complex, FANCM catalyzes fork regression permitting synthesis of an Okazaki fragment followed by recoupling of lagging and leading strand synthesis and FANCM degradation once the single-stranded DNA signal disappears and Chk1 is de-activated (Figure 8).

In a more recent study, Singh *et al.* reported that FANCM phosphorylation at Ser-1045 induced by replication stress occurs in an ATR-dependent manner, resulting in the recruitment of FANCM to ICLs, prevention of premature entry into mitosis, and efficient Chk1 and G2-M checkpoint activation [59]. However, it was not determined if ATR directly phosphorylates FANCM or if the FANCM Ser-1045 phosphorylation affected FANCM stability or its ability to translocate on double-stranded DNA to sites of blocked replication forks; nonetheless, it is apparent that FANCM is targeted by checkpoint kinases ATR, Chk1, and Plk1 which exert dramatic effects on FANCM stability and function that are important in modulating its roles in chromosomal stability and replication restart. Molecular studies that assess the biochemical effects of FANCM post-translational modifications such as phosphorylation on its catalytic activities and protein interactions will be informative for understanding its role in the FA pathway and DNA damage checkpoints.

## 8. Proteolysis of FBH1 Helicase is Managed by Its Interaction with PCNA

**Figure 9:**
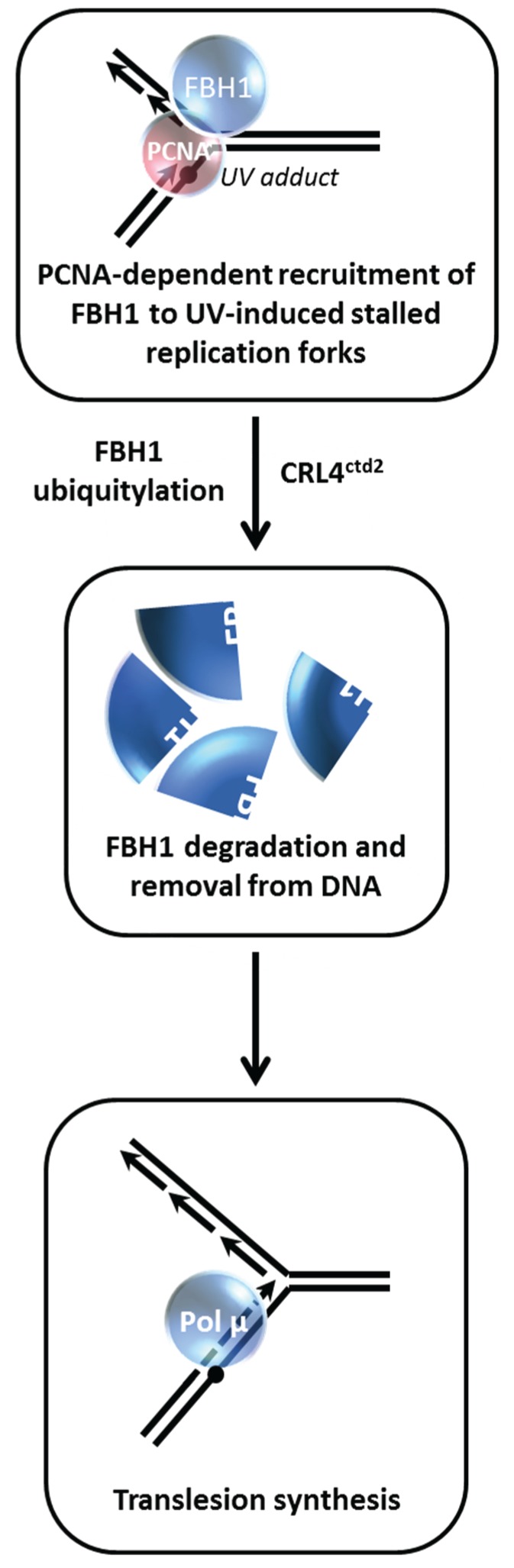
FBH1 proteolysis is governed by its interaction with PCNA during the UV-induced DNA damage response. See text for details.

Controlling repair of DSBs by modulating the functions of DNA helicases is a theme that is highly prevalent for maintenance of genomic stability [2]. The human F-box DNA helicase (FBH1) is known to regulate HR that might occur at stalled replication forks [60,61]. Bacquin *et al.* found that while PCNA serves to recruit FBH1 to sites of DNA replication and DNA damage *via* a direct physical interaction and facilitates its anti-recombinase activity, degradation of FBH1 is enhanced after ultraviolet (UV) light irradiation *via* the E3 ubiquitin ligase Cullin-ring ligase 4-Cdt2 (CRL4^Cdt2^) in a manner that is dependent on FBH1’s interaction with PCNA [26] (Figure 9). The authors proceeded to show that expression of a non-degradable FBH1 mutant diminished recruitment of the translesion polymerase Pol η to chromatin in UV-irradiated cells. This leads to the scenario in which FBH1 degradation *via* CRL4^Cdt2^-PCNA is necessary so that FBH1 can be removed from the site of the replication-blocking lesion so that the TLS polymerase can gain access and perform bypass DNA synthesis. Although it may seem antithetical, the degradation of FBH1 is necessary to insure PCNA-dependent balance of HR and TLS in human cells.

Further studies have substantiated the importance of a specialized PCNA-interacting motif (PIP box), designated the PIP degron, to target PIP-degron-containing DNA damage response proteins for proteasomal degradation by CRL4^Cdt2^ allowing a switch of PCNA partners [62,63]. However, PCNA-interacting helicases besides FBH1 have not yet been identified to be regulated in this manner. The RecQ helicase BLM may be a good candidate for PCNA-dependent regulation in a fashion similar to that of FBH1, given the dynamics of BLM degradation which are controlled by its protein partnerships. Interestingly, the human RECQL4 helicase defective in the chromosomal instability and premature aging disorder Rothmund-Thomson syndrome was found in a complex with the ubiquitin ligases UBR1 and UBR2 that are implicated in protein proteolysis; however, RECQL4 protein was determined to lack ubiquitylation and be relatively stable in unstressed HeLa cells, suggesting some other role for its interaction, perhaps subcellular localization [64]. It may be informative to assess RECQL4 degradation in cells exposed to a DNA damaging agent because replication stress elicits a cellular response that affects stability of the ATP-dependent motor translocase FANCM, which like RECQL4 [65], plays a role in replication dynamics [57].

## 9. Acetylation of Werner Syndrome Helicase (WRN) Regulates Its Stability *via* Inhibition of Ubiquitylation

**Figure 10:**
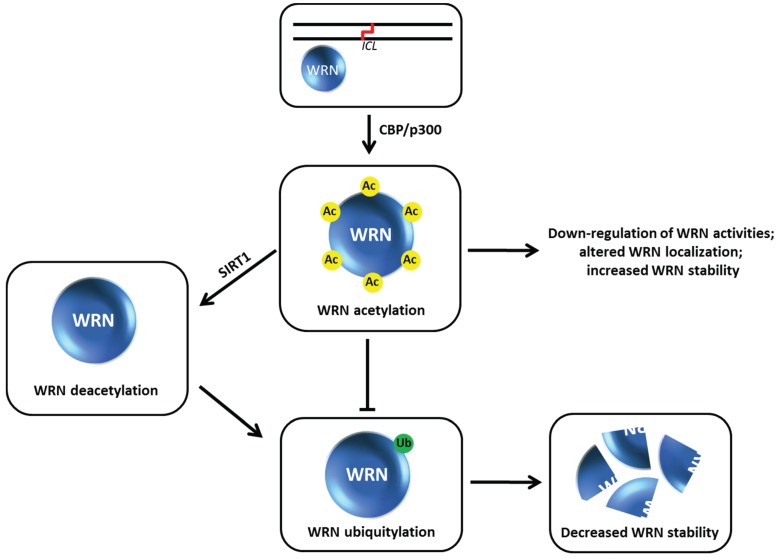
Werner syndrome helicase-nuclease (WRN) acetylation affects its stability, catalytic functions, and subcellular localization. See text for details.

The *WRN* gene, mutated in the premature aging disorder Werner syndrome, encodes a protein with both DNA helicase and exonuclease activities (for review, see [66]). Work from the Orren and Luo laboratories showed that WRN can be targeted for acetylation by the acetyltransferases CBP and p300 which down-regulates its catalytic functions and affects its subcellular localization [67] (Figure 10). WRN acetylation at six lysine residues also increases its protein stability, whereas deacetylation of WRN by the sirtuin SIRT1 destabilizes WRN [68]. WRN stability is influenced by the ubiquitin pathway, which is negatively regulated when WRN is acetylated. After cellular exposure to the DNA cross-linking agent MMC, WRN is markedly acetylated leading to its stabilization. Cells expressing a WRN variant that is mutated at the six lysine residues thereby blocking its acetylation are hypersensitive to MMC, suggesting that an important aspect of WRN’s role in the DNA damage response is its regulation by acetylation which prevents its proteolytic degradation *via* a ubiquitin-proteasome pathway. Thus WRN acetylation and deacetylation may serve as a switch during DNA damage conditions that influences WRN catalytic function or its own degradation which in turn alters its role at stalled replication forks or sites of DNA damage [69]. In the future, it will be valuable to assess if certain histone deacetylase (HDAC) inhibitors currently used in the clinic might affect the acetyltransferase that modifies WRN as it is known that this class of compounds can alter the acetylation state and function of non-histone proteins as well as their traditional histone targets [70,71,72]. Given that certain HDAC inhibitors are approved or in clinical trials for cancer treatment, it will be informative to assess if WRN-dependent pathways are involved in anti-proliferation. This may be relevant to the development of WRN helicase inhibitors for anti-cancer therapy [73]. For example, a small molecule WRN helicase inhibitor that sensitizes FA-deficient cancer cells to MMC has implications for chemotherapy strategies [74,75].

## 10. Choice of Post-Replication Repair Pathway Mediated by HLTF Degradation

Replication fork stalling at unrepaired DNA lesions can be overcome by TLS or template switching using an undamaged sister chromatid and/or HR to facilitate indirect bypass of the lesion [76]. In either scenario, the lesion is corrected after the resumption of DNA synthesis. The mechanisms of post-replication repair (PRR) have attracted considerable interest because recent evidence suggests that they appear to be dependent on the type of lesion encountered by the replication machinery. While considerable research in the genetic requirements for PRR has been done in yeast [77], only recently have the pathways and proteins involved in mammalian PRR been studied. An important player is the mammalian helicase-like transcription factor (HLTF). Human HLTF is an orthologue of yeast Rad5 which is implicated in error-free replication past DNA lesions [78]. In addition to its ubiquitin ligase activity [78], HLTF possesses the conserved helicase motifs and uses the energy of ATP hydrolysis to translocate double-stranded DNA [79]. Biochemical studies with purified recombinant protein suggest that HLTF can remodel proteins bound to DNA [80] and perform strand invasion to create a displacement (D)-loop intermediate that helps to repair gaps formed at the sites of damaged DNA during replication [81].

**Figure 11:**
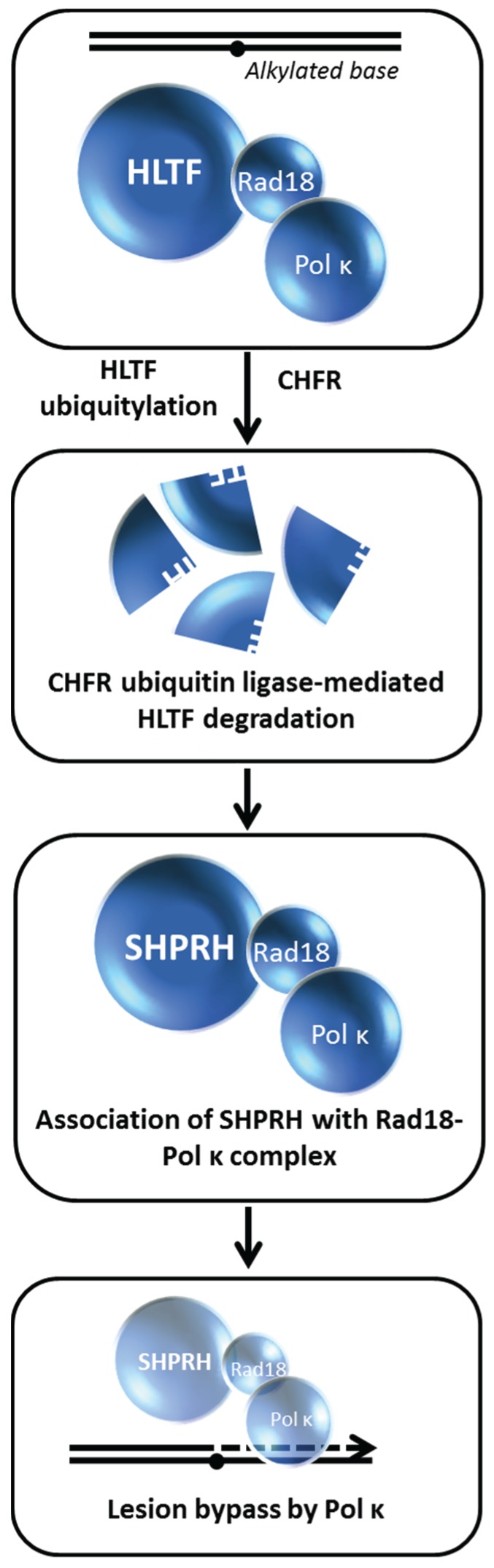
HLTF degradation enables translesion polymerase bypass of alkylated DNA damage. See text for details.

Lin *et al.* performed studies with human cells to delineate the role of HLTF from another human Rad5-related protein known as SNF2 Histone-linker PHD-finger RING-finger Helicase (SHPRH) [82]. Loss of SHPRH or HLTF causes chromosomal instability after cellular exposure to DNA damaging agents; furthermore, both proteins poly-ubiquitylate the mono-ubiquitylated form of PCNA in a manner that is important for damage avoidance. In a clever series of experiments, the authors distinguished the mechanisms whereby HLTF and SHPRH operate in a damage-specific manner, *i.e.*, HTLF suppresses UV light-induced mutagenesis and SHPRH suppresses methylmethanesulfonate (MMS)-induced mutagenesis. Cellular exposure to the alkylating agent MMS causes HLTF to be ubiquitylated and degraded in a proteasome-dependent manner, thereby promoting SHPRH to interact with Rad18 and polymerase kappa. Thus, the MMS-induced degradation of HLTF facilitates a pathway choice mediated by SHPRH because otherwise HLTF would compete with SHPRH for Rad18 binding (Figure 11). On the other hand, UV exposure causes HLTF to enhance PCNA mono-ubiquitylation, polymerase eta recruitment, and inhibition of SHPRH function. In either scenario, the recruitment of the most appropriate TLS polymerase to a specific replication blocking lesion enables an optimal bypass mechanism of PRR to minimize mutagenesis, presumably by ensuing fork progression thereby preventing fork stalling, fork collapse, and gross chromosomal rearrangements. The balancing act between HLTF and SHPRH is likely to play a paramount role in their tumor suppressor functions as they operate to suppress mutagenesis in a damage-specific manner.

In another study, a RING type E3 ubiquitin ligase known as checkpoint protein with FHA and RING finger domains (CHFR) was found to interact with HLTF and ubiquitylate it, resulting in HLTF proteasomal degradation [27]. The tumor suppressor role of CHFR was suggested to be a consequence of its ability to cause degradation of HLTF and protect against HLTF-mediated cell migration by modulating HLTF’s regulation of expression of plasminogen activator inhibitor-1 (PAI-1) implicated in tumor invasion and metastasis. Collectively, these findings suggest that HLTF stability may influence carcinogenesis by modulating gene expression control or damage-induced mutagenesis.

Consistent with the findings that ubiquitylation of HLTF causes its degradation, Qing *et al.* determined that in A549 adenocarcinoma human alveolar basal epithelial cells the deubiquitylating enzyme USP7 binds and deubiquitylates HLTF after DNA damage or replication stress induced by etoposide, HU, or MMS, resulting in HLTF stabilization [83]. Consequently, USP7 promotes HLTF-induced PCNA poly-ubiquitylation, an important signaling event to maintain genomic stability. In USP7-depleted cells, addition of the proteasome inhibitor MG132 restored HLTF levels indicating a possible role of USP7 to protect HLTF from proteasome-mediated degradation. The biological importance ascribed to the stabilizing effect of USP7 on HLTF is illustrated by the finding that HLTF over-expression in USP7-depleted cells restores cellular resistance to etoposide-induced apoptosis. Thus, UPL7 regulates HLTF’s function in DNA damage resistance by preventing its degradation *via* a ubiquitin-proteasome pathway.

**Figure 12:**
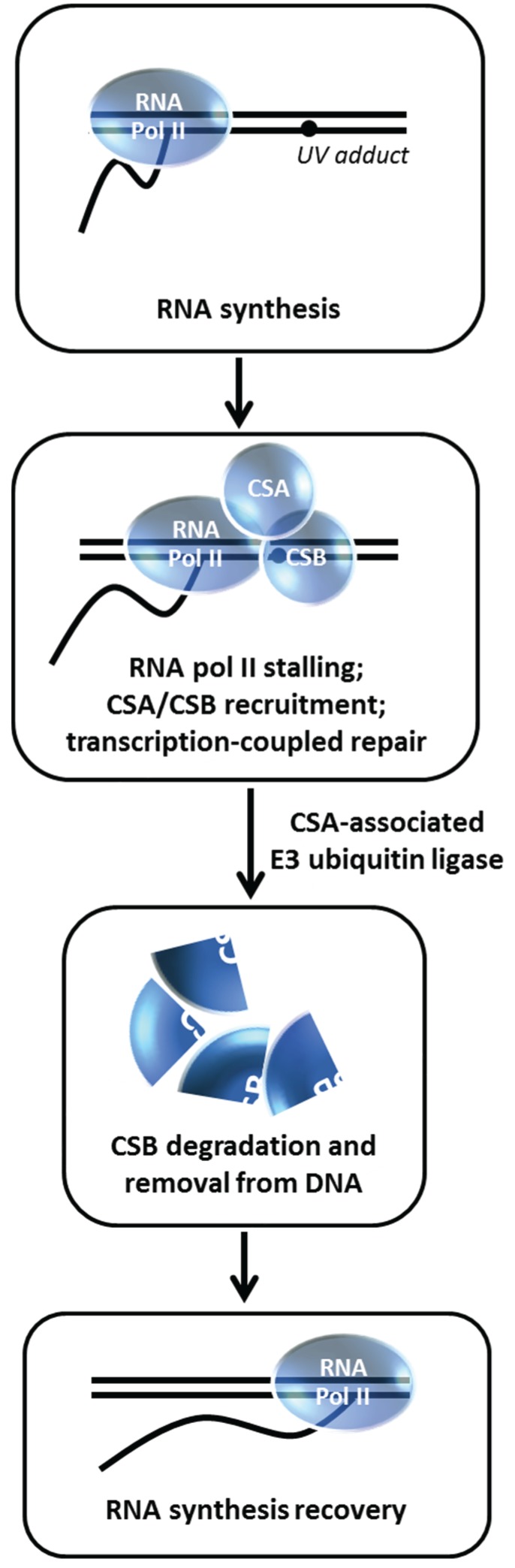
CSA-associated ubiquitin ligase insures CSB degradation enabling RNA synthesis recovery in the transcription-coupled repair pathway. See text for details.

## 11. Degradation of Cockayne Syndrome Protein CSB by CSA-Mediated Pathway is Important for Post-Repair Recovery of RNA Synthesis

The DNA repair disorder Cockayne syndrome (CS) is an autosomal recessive disease caused by mutations in the *CSA* or *CSB* genes [84]. The *CSA* gene encodes a WD-repeat protein that is associated with Cullin 4A (CUL4A) E3 ubiquitin ligase. The *CSB* gene encodes a SWI2/SNF2 DNA-dependent ATPase that can remodel chromatin. Cells derived from CSA or CSB patients are UV-sensitive and defective in transcription-coupled repair (TCR), resulting in delayed cover of RNA synthesis after UV-induced DNA damage. Understanding the precise molecular role(s) of CSA and CSB in TCR has posed a daunting challenge in the field. It is generally believed that the initiating signal for TCR is a stalled RNA Polymerase II at the lesion, followed by recruitment of CSA and CSB which are required for further assembly of nucleotide excision repair (NER) proteins responsible for removal of the lesion [84]. Groisman *et al.* discovered a functional relationship between CSA and CSB with the demonstration that CSB is degraded by an E3 ubiquitin ligase complex that contains CSA [28] (Figure 12). Normally, CSB is degraded within 4 h post UV irradiation in a proteasome-dependent manner; however, in CSA-deficient fibroblasts, CSB level is unaffected after UV exposure. *In vitro* studies demonstrated that the CSA-associated E3 ligase complex directly ubiquitylates CSB. The authors next wanted to assess the biological importance of CSB degradation post UV treatment. Building on an earlier observation that cellular exposure to a proteasome inhibitor suppresses RNA synthesis recovery [85], Groisman *et al.* determined that CSB persists in the CSA complex in MG132-treated cells and that the residual RNA synthesis recovery in CS-A or CS-B cells is insensitive to MG132 [28]. Thus, CSB degradation mediated by the CSA E3 ubiquitin ligase complex is necessary to remove CSB from the DNA after it has fulfilled its role to recruit NER proteins so that transcription can recover at the repaired site. In a more recent study, evidence was presented that BRCA1 poly-ubiquitylates CSB protein leading to CSB degradation and resulting in improved UV resistance, suggesting a pathway in addition to the CSA-dependent one [29].

## 12. Conclusions

Degradation of DNA helicases or helicase-like proteins serves important functions in nucleic acid metabolism. From the examples presented here, the biological importance is revealed in distinct ways: (1) BLM: SCE suppression, regulation of HR repair, and replication restart; (2) FANCM: fork regression and Chk1 activation; (3) FBH1: translesion DNA synthesis; (4) HLTF: post-replication DNA repair; (5) WRN: cross-link resistance; (6) CSB: RNA synthesis recovery after DNA damage repair. Thus, the importance of degradation of DNA helicase or helicase-like proteins is manifest by distinct pathways that largely involve post-translational modifications and protein interactions. These helicase degradation mechanisms, most often mediated by ubiquitin proteasome complexes, are frequently elicited by cellular responses to DNA damage or replication stress. Many of these degradation pathways are essential for DNA repair, maintenance of chromosomal stability, and/or transcription recovery. Modulation of DNA helicase degradation pathways may present an avenue for anti-cancer therapies directed toward improving the efficacy of chemotherapeutic DNA damaging drugs or radiation.
